# Trajectories of emigrant quasi-citizenship: a comparative study of Mexico and Turkey

**DOI:** 10.1186/s40878-017-0061-3

**Published:** 2017-11-24

**Authors:** Rusen Yasar

**Affiliations:** Department of Politics and Public Administration, University of Konstanz, PO Box 90 D-78457, Konstanz, Germany

**Keywords:** Plural citizenship, Quasi-citizenship, Emigration states, Citizenship constellations, Turkey, Mexico

## Abstract

In two of the busiest migration corridors of the twentieth century, namely Mexico-US and Turkey-Germany, migrants can today be dual citizens. However, the acceptance of dual citizenship did not occur automatically; instead, it followed a period of legal statuses short of full citizenship. This paper conceptualises such statuses as quasi-citizenship, a transitional equilibrium between the absence of plural citizenship and the existence of transnational migration. Focusing on sending states, the emergence of emigrant quasi-citizenship is thus explained, first, in terms of whether the reciprocal regimes of emigration and immigration states diverge on the acceptance of plural citizenship. Second, the stance towards plural citizenship is explained in terms of the experience with emigration. It is then shown that, in the case of Mexico, the legacy of undesired emigration weakened the incentives to adapt the territorial conception of citizenship to expatriates, hence creating quasi-citizens, and in the case of Turkey, the higher political relevance of expatriates, who could have the host country citizenship, reinforced the external dimension of the ethno-cultural conception of citizenship.

## Introduction

Two of the busiest migration corridors of the twentieth century were between Mexico and the US, and Turkey and Germany. Although migrants and their descendants now have the possibility to possess dual citizenship in both cases, migration and its implications are still a politically contentious issue. For example, the last year witnessed the controversial electoral campaign of now president Donald Trump, in which migration from Mexico was a central topic, and the quick escalation of a diplomatic crisis between the Turkish government and several European states, because of the former’s insistence to extend their campaign for a constitutional referendum towards Turkish communities living in Europe. Such controversies are also likely to have repercussions for citizenship regimes, as the relatively easy acquisition of the US citizenship and the dual loyalty of Turkish-origin migrants are being questioned. In the light of such recent developments, the aim of this paper is to shed light on the recent history of the development of migration and citizenship policies, which has led to the current positions of involved actors and institutions. Furthermore, the regimes which permit dual citizenship may not be very stable, and their historical trajectories would probably shape any potential departure from the acceptance of dual citizenship. This is especially relevant for the Germany-Turkey nexus where dual citizenship is not deeply institutionalised, but allowed as a result of not implementing the previous requirement of choosing only one citizenship.

The main interest of the paper is in the policies and institutions of emigration states concerning their (former) citizens. Although the study of sending state policies is relatively new as compared to the study of receiving state policies, an important body of academic works has been produced with regard to emigrants of Latin American and especially Mexican origin living in the United States (e.g. Fitzgerald, 2006a,b, 2009; Goldring, 2002; Itzigsohn, 2000; Jones-Correa, 2001), while the diffusion of emigration state policies continue among Latin American countries, following in particular the Mexican model (Délano, 2014). Meanwhile, Østergaard-Nielsen (2003b,c) not only compiles a large array of case studies, hence a comprehensive overview of sending state policies, but also provides a pioneering study of Turkish state’s reaching out efforts towards emigrants. This line of research is significantly advanced since then, in particular through the contributions by (Mügge, 2012b, 2013), but the study of the implications for emigration state citizenship still remain focused on migrant practices (e.g. Mügge, 2012a) rather than home state institutions.

Therefore, comparing Turkey and Mexico is promising in view of not only the topicality of the cases, but also their potential contribution to migration and citizenship studies. Earlier studies of these cases, led by Faist (1992, 1994, 1995, 1996), testify to the usefulness of the comparison for explaining the relationship between political systems, citizenship regimes and immigration and integration policies of the receiving states, as well as the rights possessed and socio-economic positions occupied by immigrants. Nonetheless, this potential has not been sufficiently exploited since then, especially with regard to emigration state policies, although Mexico and Turkey represent an analytically interesting contrast against the background of broad similarities of being labour-exporting emigration countries. Namely, Mexico resisted for a long time the global trend towards plural citizenship in the face of a permissive American regime, and Turkey has promoted plural citizenship for a long time although Germany was reluctant. As a result, the last restrictions against plural citizenship were removed by Mexico in 1997 as part of a legislative reform and by Germany as part of the coalition government formed in 2013. In this respect, this paper explains why such a contrast occurs, and why the transition to effective plural citizenship is concluded by the sending state in one case and by the receiving state in the other.

In this context, the focus will be on relatively long periods of transition that precede the acceptance of plural citizenship. The formal categories pertaining to migrants or migrant-origin communities in such periods, when dual citizenship was not introduced by all parties, will be conceptualised as quasi-citizenship. There is not a standard definition of the term ‘quasi-citizenship’ in the literature; it can be used to refer to denizens or long-term residents (Nash, 2009), to those who enjoy a more enhanced status than denizens (Groenendijk, 2006), or to co-ethnic communities living in a different country (Knott, 2017). Furthermore, Bauböck (2006, p. 2396; 2010, pp. 848–849) uses the term rather broadly, to qualify both long-term residents and external co-ethnic communities. In a similar way, quasi-citizenship will be conceptualised broadly in this paper, as statuses less extensive than full citizenship but more extensive than being a regular alien, while the focus for its external aspect will be on emigration contexts. Accordingly, emigrant quasi-citizenship may occur when the sending state pushes the overall equilibrium towards dual citizenship, as exemplified by the case of Turkey, or when the sending state pulls the equilibrium away from dual citizenship, as exemplified by the case of Mexico. As to why two similar emigration states display such diverging preferences, the paper comparatively reviews the main characteristics of their citizenship regimes and emigration histories.

It is argued that the pre-existing conceptions of citizenship and the specific features of migration interact to shape the trajectories of emigrant quasi-citizenship. On one hand, Mexican citizenship regime is based on a territorial conception, which required a more fundamental change for extending full citizenship to emigrant communities. Yet the incentives for such a change were also weakened by the unclear benefits of recognising dual citizenship, especially given the historical legacy of undesired emigration which had occurred when population outflow was demographically and economically harmful for the country. On the other hand, Turkish citizenship regime is based on an ethno-cultural conception, which is compatible with recognizing emigrant communities as full citizens. Moreover, the political benefits of the existence of dual citizens in Europe strengthens the incentives to sustain formal ties with them.

The remainder of the paper is structured as follows. The first section develops the theoretical framework by conceptualising quasi-citizenship and describing the comparative logic of the empirical sections. The second section reviews the citizenship regimes of Mexico, the US, Turkey and Germany to describe the forms of quasi-citizenship under scrutiny and their relationship to different conceptions of citizenship. The third section reviews the emigration histories of Mexico and Turkey in order to determine the differences which explain their diverging preferences. The fourth section discusses the empirical findings and the last section concludes.

## Transnational migration and quasi-citizenship

### Conceptualising quasi-citizenship

According to a unitary conception of citizenship, citizenry would be a group of people living in a well-defined territory as equal members of a political community and subject to the same political authority. However, in the context of transnational migration, such an overlap no longer exists, with the presence of non-citizens in the territory and under the authority of immigration states, and with the absent citizens outside the territory and beyond the reach of the political authority of emigration states. Therefore, transnational citizenship can be understood as resulting from the non-congruence between geographic and social/political spaces that made nation-building and national citizenship possible (Pries, 2000; Smith & Guarnizo, 1998). Thus migrants ‘build social fields that link together their country of origin and their country of settlement’ (Schiller, Basch, & Blanc-Szanton, 1992, p. 1), leading to ‘overlapping memberships between territorially separated and independent polities’ (Bauböck, 2003, p. 720). However, the analytical value of the category of ‘transnational citizenship’ can be easily compromised if its use leads to the conceptual stretching of the broader category of ‘citizenship’ (Fox, 2005). Since the only viable use of transnational citizenship seems to be the cases of plural citizenship, there is a need for a concept which explicitly acknowledges the differences of content between full citizenship and other legal statuses similar to it. The concept of quasi-citizenship is proposed here for this purpose.

These grey areas constitute the context from which quasi-citizenship emerges. The emergence of a new category less extensive than full citizenship can be explained by the idea that the main components of citizenship have been disarticulated from each other, and partially rearticulated in transnational spaces (Benhabib, 2007; Cohen, 1999; Ong, 2006). These components can be taken in terms of the dimensions of status, rights and identity (Joppke, 2007; Kymlicka & Norman, 2000)^1^. The status dimension refers to the expression of legal ties between the individual and the state, thus, both an instrument and the object of closure that creates boundaries between people and allocates them to distinct entities (Brubaker, 1994). Given transnational spaces, the physical distribution of people will not match such a legal allocation. The identity dimension refers to the expression of social ties which may display both civic and cultural components. Yet the cultural dissimilarity created by immigration, and cultural similarity becoming the most obvious link with expatriates, lead immigration states to de-emphasise and emigration states to re-emphasise ethnic aspects of citizenship (Joppke, 2003).

As for the dimension of rights, this can be further disaggregated into three components, following the seminal essay by Marshall (1965): civil, political and social rights. For those who take rights as the most crucial dimension, it is possible to argue that national citizenship has become obsolete since rights are now attached to universal personhood rather than membership in a nation state (Soysal, 1994). Nonetheless, it is difficult to sustain this claim for all generations of rights. While it is true that civil rights are near-universal, and that social rights are more or less detached from citizenship depending on the system of welfare state, political rights are still almost exclusively linked to citizenship^2^. In this sense, due to the disarticulation and rearticulation of rights among themselves, transnational spaces may witness different configurations of rights, and the presence and absence of political rights is usually the crucial feature which demarcates full citizenship from other categories. Furthermore, political rights are not necessarily entailed directly from status; although, in general, naturalisation is a condition for immigrants, external voting requires additional arrangements for the materialisation of emigrant political rights, which may display large variation across countries (Nohlen & Grotz, 2007).

In this framework, the content of quasi-citizenship becomes a question of which components of full citizenship, including different types of rights, are articulated to define new categories of people. Table 1 provides a summary of several possibilities between the categories of traditional citizen and alien, with regard to the components of citizenship as well as residential status, without any claim of ordering in between. To begin with the first category according to this scheme, the only difference of a citizen with immigrant background from a traditional citizen would be cultural identity, which is increasingly losing its relevance as mentioned above. In other words, the category of immigrant-origin citizen is not fundamentally different from the traditional citizen, especially if cultural identity is replaced by a more civic link between the individual and the state. However, the distinguishing feature of the emigrant-origin citizen is residence, which is difficult to underplay even in the age of communication and convenient transportation. In this sense, the legal status of emigrants remains a qualitatively different and analytically interesting category.

**Table 1:** Combinations of the components of citizenship

	Membership	Civil rights	Political rights	Social rights	Cultural identity	Residence
Traditional citizen	Yes	Yes	Yes	Yes	Yes	Yes
Immigrant-origin citizen	Yes	Yes	Yes	Yes	No	Yes
Emigrant-origin citizen	Yes	Yes	Yes	Yes	Yes	No
Enfranchised denizen	No	Yes	Yes	Yes	No	Yes
Ordinary denizen	No	Yes	No	Yes	No	Yes
Emigrant quasi-citizen 1	Yes	Yes	No	Yes	Yes	No
Emigrant quasi-citizen 2	No	Yes	No	Yes	Yes	No
Alien	No	Yes	No	No	No	No

In spite of such differences, the first three rows of Table 1 represent full citizenship. Thus, quasi-citizens can be understood as those who differ from these three categories in at least one other dimension. The permanent residents of a country who have not acquired its citizenship, also known as denizens (Hammar, 1990), would thus constitute one group of quasi-citizens, as they do not have the basic legal status. Moreover, with the exception of a few countries which attach certain political rights to residence, they would normally not be enfranchised. The main interest of this paper is the next two categories of emigrant quasi-citizens. The first category retains a legal status but loses the political rights due to not being a resident of the country of origin any more, and this is exemplified by the case of Mexico. The second category does not retain this status but enjoy certain rights and privileges not recognised to aliens, and this is exemplified by the case of Turkey.

At this point, a few qualifications should be added to this categorisation. First, the idea of social rights is used quite broadly in Table 1, as they are enjoyed by all categories but the alien. In this broad understanding, they may include exemptions from the restrictions on property and inheritance which may normally apply to aliens. Since all categories of people are assumed to benefit from universal human rights, the main feature distinguishing the second category of emigrant quasi-citizens from aliens is such differential treatments due to cultural or historical ties between the emigrants and the country of origin. Second, the conceptualisation of quasi-citizenship as above is intended as distinct from the ‘lightening of citizenship’ (Joppke, 2010): it is concerned with the differences between full citizenship and less extensive formal statuses, even if the relevance of the former is diminishing over time. Third, this categorisation is not necessarily exhaustive. For example, the relationship between an undocumented immigrant and the country of destination could not be captured by any of the above categories, or there might be countries which do not offer any special status to emigrant non-citizens. Fourth, only migration-related categories are used here, although the concept of quasi-citizenship can also apply to other transnational relations, such as those with kin communities (Knott, 2017). This type of quasi-citizenship, also framed as ‘ethnizenship’, would contain the same components as the second category of emigrant quasi-citizenship here, but it would still be a qualitatively different category.

In this context, this paper will try to answer the broad question as to how such intermediate categories between citizens and aliens emerge and disappear. More specifically, what prevents immigration and emigration states from allowing plural citizenship at a certain point in time, resulting in various forms of quasi-citizenship, and what leads them to accept it at another point in time, making the categories of quasi-citizenship redundant? As noted before, the focus of this paper is on emigration states, and in this sense, the following section will offer a basic model regarding how they respond to the conditions set by immigration states.

### Two trajectories

At a broader level, the emergence and the disappearance of emigrant quasi-citizenship, and its different forms, can be explained by the multiplicity of actors, and the divergence and convergence of their preferences. One implication of transnational migration is that political decisions regarding citizenship can no longer remain entirely domestic; in other words, one should look at ‘citizenship constellations’ comprising of at least the sending state, the receiving state and the migrant communities (Bauböck, 2010). In this case, the appropriate starting point to understand the citizenship statuses of the migrant communities will be the reciprocal positions of the two states. Table 2 offers a simple summary of what might result from preferences regarding single and plural citizenship regimes.

**Table 2:** Reciprocal positions of states regarding plural citizenship

		Receiving state preference:	
		Single	Plural
Sending state	Single	(I) Agreement on single citizenship	(II) Disagreement: quasi citizenship 1
preference:	Plural	(IV) Disagreement: quasi-citizenship 2	(III) Agreement on plural citizenship

Given the global trend towards the acceptance of plural citizenship (Martin, 2003; Pogonyi, 2011; Spiro, 2007), the position (III) can be expected to represent the ultimate equilibrium. More specifically, the actors would start in position (I) in a world where transnational migration has not far-reaching effects; with the rising significance of transnational migration, either the sending state or the receiving state alters its preference in favour of plural citizenship; whether the sending state or the receiving state makes the first move determines the trajectory through position (II) or (IV); when the other actor also alters its preference, a stable equilibrium in position (III) is reached.

Nonetheless, the model should account for two important specificities that emerge from the historical trajectories of migration and the resulting regimes. First, there are large time differences between the waves of migration and the eventual acceptance of plural citizenship, which means relatively lengthy transition periods. In this sense, quasi-citizenship can be understood as a transitional equilibrium rather than non-equilibrium. Second, plural citizenship is not always deeply institutionalised even though it is effectively practised, as in the case of Germany, which means a relatively less stable equilibrium. Therefore, a setback from the agreement on plural citizenship is possible, and if it occurs, the citizenship regime would once again end up in a transitional equilibrium of quasi-citizenship. For these reasons, the study of quasi-citizenship constitutes a crucial part of understanding the overall transition between single and plural citizenship regimes.

Two main implications follow when the above framework, the general observation about the trend towards plural citizenship and the special importance of transitional equilibria are considered together. First, emigrant quasi-citizenship can be broadly explained as resulting from a discrepancy between the preferences of sending and receiving states. Moreover, the type of quasi-citizenship may be determined by reciprocal positions of the states; with this anticipation, the first and second categories of emigrant quasi-citizenship as depicted in Table 1 are tentatively placed into the positions (II) and (IV) of Table 2 respectively. Although inductive inference from the cases of Mexico and Turkey certainly plays a role in this anticipation, a more theoretical explanation is also possible.

The underlying assumption here is that each state uses its own sovereign prerogatives to affect the citizenship constellation, since it is less likely to have a considerable leverage on the preference of its counterpart. In this case, a sending state, which prefers plural citizenship but cannot convince its respective receiving state to the same, would push the equilibrium towards plural citizenship by other means. Namely, it can offer certain privileges to former citizens to facilitate the acquisition of the receiving state citizenship and retaining formal ties with emigrants at the same time. On the other hand, a sending state, which prefers single citizenship but cannot easily stop the receiving state to grant a second citizenship to its emigrants, would push the equilibrium away from plural citizenship by other means. Namely, it can curtail the content of the status owned by the emigrants to ensure that potential dual citizens cannot enjoy the full benefits of regular citizenship.

The second implication of the above model is concerned with the explanation as to why actors have different preferences regarding single or plural citizenship, which eventually lead to different possibilities of citizenship constellations. According to the proposed account, the movement from the single citizenship constellation towards the plural citizenship constellation is triggered by the rising importance of transnational migration. In this sense, the pace at which the transition is completed, or the period which corresponds to quasi-citizenship, can also be a function of the transnational migration. Therefore, the cases in which transnational migration is more important, or recognised as such, should be the cases where the tendency to move towards plural citizenship is stronger. In order to test this hypothesis, a more empirically oriented discussion will be necessary. For this reason, the remainder of this paper will be built on a comparative study, and examine in detail the citizenship regimes and emigration histories in the constellations of Mexico-US and Turkey-Germany.

### Comparative framework

What makes a comparison of Mexico and Turkey interesting is the historical divergence in their preference for plural citizenship, hence the concomitant forms of emigrant quasi-citizenship, against the background of broad similarities. This implies a typical ‘most similar systems’ design (Lijphart, 1971; Przeworski & Teune, 1970; Skocpol & Somers, 1980), whereby a small number of variables which display differences across cases are inferred to be associated with each other. However, perfectly similar cases are impossible to find, and this study cannot claim to be exhaustive about similarities and differences. Instead, Turkey and Mexico are claimed to be similar and different to a sufficient extent and belonging to a certain class of cases; inferences can thus be made to this class (Sartori, 1991). Following Sartori’s (1970) ‘ladder of abstraction’ logic, such inferences can be made more confidently when the inferred class of units is more restrictively defined to correspond to the studied cases, and less confidently when it is more broadly defined.

In this sense, it is worth discussing what Turkey and Mexico are cases of, and which inferences can be more confidently derived from their comparison. The category of ‘emigration states’ could be defined as largely as diaspora-like relations (Bauböck & Faist, 2010; Østergaard-Nielsen, 2003b) which would include ancient spreading of people from a homeland (e.g. Jewish communities around the world), legacy of colonial empires (e.g. Indian community in the UK), result of historical tragedies (e.g. Armenian communities in Western Europe and Americas), or old migration flows (e.g. Italian community in the US), among others. Yet Turkey and Mexico are more appropriately understood in terms of more recent waves of migration, intensifying in the twentieth century and taking the shape of labour export from peripheral (low or middle income) countries to advanced industrial economies; generalisations beyond this category to other types of diaspora relations should be made with caution and take into account the differences between such types. Furthermore, the migration from Mexico and Turkey concentrated into single destinations, the US and Germany respectively^3^, as the above discussions in Two trajectories section also modelled. This simplifies the analysis since sending states can be safely assumed to be adjusting their policies in view of only one receiving state regime. While the findings of this study will probably be relevant for multi-destination cases to a certain extent as well, it should be remarked that a more complex analysis would be necessary.

As for the content of comparison, the main variable that displays difference across cases and that this paper tries to explain is the type of emigrant quasi-citizenship. In view of the model proposed in “Two trajectories” section, the degree to which plural citizenship can be accommodated in the reciprocal citizenship regimes of sending and receiving states will constitute the first stage of analysis. This will be followed by an explanation as to why sending states differ in their preference for or against plural citizenship, in terms of their emigration experiences. Since both cases broadly have a similar history of emigration as labour export, a detailed examination will be necessary to observe the differences, and a variety of variables will be considered for this purpose.

An overview of the literature on sending states (e.g. Brand, 2006; Itzigsohn, 2000; Smith, 2003a) reveals factors including domestic politics of the home country, its position within the global economic system and international politics, the situation of migrants in their country of residence, and direct economic benefits. Yet there may exist a variety of incentives for sending states to reach out to emigrants, not limited to simple economic benefits but including expectations related to international political aims (Østergaard-Nielsen, 2003a). Meanwhile, the emigrants must not be taken as passive recipients of sending state policies; not only their actorness must be recognised but also special attention should be paid to the interactive nature of their relationship with home countries (Barry, 2006). Such factors will be examined in two groups: first, the historical context of emigration covering demographic, economic and political pressures, and second, the development of sending state policies covering the economic and political incentives, perception of emigrants, emigrants’ interest in home country and the institutionalisation of relations. Together with the factors related to citizenship regimes, Table 3 lists the variables to be comparatively discussed and summarises the findings which will be presented in the sections below.

**Table 3:** Summary of comparison

	Mexico	Turkey
*Citizenship regimes*	*Mexico-US constellation* * section*	*Turkey-Germany constellation* * section*
Quasi-citizenship	Pulls the equilibrium away from dual citizenship	Pushes the equilibrium towards dual citizenship
Emigration state regime	Territorial	Ethno-cultural
Immigration state regime	US: Long tradition of liberal regime	Germany: Late and partial liberalisation
*Historical context of emigration*	*Demographic, economic and political pressures in Mexico* * section*	*Demographic, economic and political pressures in Turkey* * section*
Demographic pressures	Initially underpopulated	Overpopulated
Economic pressures	Compatible with development objectives	Compatible with development objectives
Political pressures	Exit as a safe option for opposition	Exit as a safe option for opposition
*Sending state policies*	*Incentives, perceptions and demand for emigrant incorporation in Mexico* * section*	*Incentives, perceptions and demand for emigrant incorporation in Turkey* * section*
Economic incentives	Rising importance of remittances	Declining importance of remittances
Political incentives	Modest importance of regional integration, intensifying in the 1990s	High importance of regional integration, sustained over time
Perception of emigrants	Contempt for cultural dissimilation	Contempt for cultural dissimilation
Emigrants’ interest	Little or modest interest in home politics	Persistence of home politics in the country of residence
Institutionalisation of relations	Central efforts of coordination	Central efforts of coordination

The comparison thus confirms that the experience of sending states with migration is related to their preference for or against plural citizenship. In this sense, Mexico is an actor that pulls the equilibrium away from dual citizenship, not only because its citizenship regime is based on a territorial conception which is not perfectly compatible with absent citizens, but also because any incentive to reform this regime could be weakened by the legacy of undesired emigration, which was harmful when the country was facing demographic challenges, and the low relevance of citizenship for expected economic benefits. On the other hand, Turkey emerged as an actor which pushed the equilibrium towards dual citizenship, not only because its ethno-cultural conception of citizenship is compatible with the endorsement of expatriates, but also because dual citizenship was crucial for the contribution of emigrants to international political goals.

## Citizenship constellations

### Mexico-US constellation

#### Citizenship regime of Mexico

The current version of the constitutional definition of Mexican citizenship is codified in Article 30 in 1997, at the same time with the acceptance of plural citizenship:

I. Those born on the territory of the Republic, regardless of the nationality of their parents. II. Those born abroad, children of Mexican parents born in the national territory, of a Mexican father born in the national territory, or of a Mexican mother born in the national territory. III. Those born abroad, children of Mexican parents by naturalisation, of a Mexican father by naturalisation, or of a Mexican mother by naturalisation. IV. Those born in Mexican ships or aircraft, merchant or war.^4^

Assuming that potential dual citizens are those who are entitled to a foreign citizenship by virtue of being born on a foreign country, which corresponds to the situation of most emigrants in the US, dual citizenship is allowed only to those who emigrated and the first generation born abroad according to this definition. Thereby, Mexican authorities sought to allow dual citizenship but limit unconditional transmission of citizenship to further generations abroad. This choice can be explained by a strong component of *jus soli* in the conception of citizenship, which prevailed after the last amendments to the constitution. In this sense, although this reform was motivated by considerations for emigrants whose links to their home country could be more easily framed in terms of ethnicity or lineage, it did not lead to a re-ethnicised conception of citizenship (cf. Joppke, 2003).

In fact, while this version is a model of full *jus soli* complemented with restricted *jus sanguinis*, the previous version was a model of mixed *jus soli* and *jus sanguinis* with equal weight, providing the opportunity to become a citizen to anyone born on the territory and born to Mexican parents regardless of territory (Fitzgerald, 2005; Hoyo, 2015; Ramírez Becerra, 2000). Yet this was counterbalanced by the restriction on acquisition of another nationality: any Mexican national who acquired another citizenship would automatically lose Mexican nationality or anyone entitled to another citizenship would have to choose. Of course, such measures would be meaningful insofar as Mexican authorities had access to the information on other citizenships, and there was a large room for unreported plural citizenship.

Since there was no restriction on plural citizenship on the part of the US, the 1997 reform made dual citizenship legally effective. For this reason, the concept of quasi-citizenship applies to the case of Mexico mostly for the period before this reform. In this respect, the constitution makes an interesting distinction between nationality and citizenship, the former referring to the legal status only while the latter referring to the rights and duties associated with citizenship. Article 34 defines citizenship by further qualifying nationals, and grants the rights to vote and be elected to the citizens. When the entitlement to enjoy the full set of rights is conditional upon residence criteria, for instance by denying voting to expatriates, nationality alone thus become a form of quasi-citizenship, comparable to Turkish blue card which is discussed below (Faist, 2000). However, external voting was immediately implemented after the institutionalisation of plural citizenship, and this was in fact so quick that several controversies soon followed, such as that of ‘Tomato King’ who was elected to a local office without being a resident^5^. Therefore, the reform did not leave room for a quasi-citizenship in the form of expatriates who hold the Mexican nationality without political rights.

#### Citizenship regime of the US

Nowadays, the US is probably witnessing the apex of anti-migrant attitudes. However, the historical variability of immigration policies stands in stark contrast to the stability of principles and legal norms which regulate the citizenship regime (Spiro, 2015). On the one hand, it is true that the history of American immigration policy includes ethnically preferential treatment, such as the Chinese Exclusion Act of 1882 which was not abolished until World War II, qualitative and quantitative controls, and tightening legislation in the second half of twentieth century. On the other hand, the current definition of citizenship is traced back to the Civil Rights Act of 1866, constitutionalised in 1868 under Amendment XIV, according to which ‘All persons born or naturalized in the United States, and subject to the jurisdiction thereof, are citizens of the United States and of the State wherein they reside.’

Resistance and tolerance towards dual citizenship stem largely from path-dependent processes (Faist, Gerdes, & Rieple, 2004). As a result of the given definition of citizenship and its broader context, the US adopts a permissive approach to plural citizenship as well. Namely, it is historically an immigration country, naturally with a civic conception of citizenship supported by birthright acquisition as *jus soli*. The ensuing institutional practices also conform to this liberal pattern, which are visible in relatively easy naturalisation procedures. The only barrier against plural citizenship can be attributed to the Oath of Allegiance, a requirement for naturalisation, which begins with the statement ‘I hereby declare, on oath, that I absolutely and entirely renounce and abjure all allegiance and fidelity to any foreign prince, potentate, state, or sovereignty of whom or which I have heretofore been a subject or citizen.’ However, there is no formal requirement of renouncing any existing citizenship beyond this declaration, and despite this virtual incompatibility, plural citizenship has long been an unproblematic feature of the US regime (Bloemraad, 2007).

### Turkey-Germany constellation

#### Citizenship regime of Turkey

Turkish citizenship is defined in the Article 66 of the constitution by the statement that ‘Everybody linked to the Turkish state with a citizenship bond is Turk’, also codifying the birthright acquisition of citizenship through that of parents. Provisions of the Citizenship Law refers to *jus soli* only under special circumstances, and there is no restriction on *jus sanguinis* transmission abroad. With a strong genealogical component, the ethno-cultural conception of Turkish citizenship seems at odds with the republican premises of the constitution (Kasaba, 1997; Kirisci, 2000). Turkish citizenship can thus be understood in terms of its monolithic character assuming a unique identity, constituted on republican but state-centric premises, and culturally embedded in the national identity (Içduygu, Çolak, & Soyarik, 1999; Soyarik-Sentürk, 2005). While these aspects mark a continuity in the citizenship regime, major changes have resulted from this official understanding of national identity and the emerging need to address the new conditions created by emigration (Kadirbeyoglu, 2007, 2009). Therefore, the acceptance of dual citizenship is completely compatible with the general character of Turkish citizenship; given the ethno-cultural conception, a re-ethnicisation was not necessary since retaining this conception was sufficient.

Nonetheless, the implementation of dual citizenship would not be effective for emigrants living in Germany because of the latter’s insistence on single citizenship regime and renunciation requirement for both birthright acquisition and naturalisation. Although Germany was in a good position to control the fulfilment of such requirements, there was still possibility to circumvent them and possess dual citizenship. For example, when there was no restriction on the acquisition of another citizenship by those who already possess German citizenship, emigrants could become dual citizens by renouncing Turkish citizenship before naturalisation and re-acquiring it afterwards, for which Turkish authorities were being extremely helpful (Hailbronner & Farahat, 2015; Rumpf, 2003). However, German authorities closed these loopholes, and Turkish authorities had to develop legally less contentious alternatives to facilitate the acquisition of German citizenship, which led to the emergence of a new form of quasi-citizenship.

In that context, acquiring German citizenship would mean losing both official ties with Turkey and the rights associated with its citizenship. In order to deal with these disincentives, Turkish authorities invented a status of quasi-citizenship, knowns as blue (formerly pink) card, offered to those former citizens who renounced Turkish citizenship in order to acquire another one, and covering almost all rights associated with citizenship except political rights. The first formulation in the Citizenship Law created a status that could be transmitted by descent without any limit, making it almost indistinguishable from citizenship, which could have looked like, from the viewpoint of German authorities, an equivocation to rebrand Turkish citizenship and circumvent the German system. Apparently, Turkish authorities also decided that this was excessive, as they curbed its transmission to the children born before the renunciation of citizenship. Therefore, this form of quasi-citizenship remains quite minimal; not only is it lacking in a status comparable to nationality and political rights, but also it did not appeal to migrants who attach a particular importance to the identity aspect of citizenship, especially the first generation(Çaglar, 2002; Anil, 2007).

Finally, on the issue of political rights, Turkey was not able to implement effective external voting due to several legal and practical impediments, despite the willingness of the government for a while (Kadirbeyoglu, 2012). Instead, a modest version of external voting has been in place in the Turkish electoral system for quite a long time in the form of placing ballot boxes in the border zones during 70 days before elections. Notwithstanding the transition to external voting since 2014 presidential elections, it can be safely argued that Turkey did not deny voting rights to emigrants on the basis of residence requirements. Therefore, single or dual, Turkish emigrant citizens cannot be seen as quasi-citizens insofar as they hold the legal status which also entails the opportunity to exercise political rights.

#### Citizenship regime of Germany

The path-dependence of citizenship regimes is also apparent in Germany with a completely different historical legacy. The conception of German nationhood in ethno-cultural terms is reflected both in the citizenship regime and immigration policies (Brubaker, 1994; Koopmans, Statham, Giugni, & Passy, 2005). The Federal Republic used the Nationality Law of 1913 as its basis of citizenship regime; while it clearly dissociated itself from preceding practices, it also found it difficult to depart from the basic conception of nationhood and to recognise that it had become an immigration country (Klusmeyer, 2009). The resulting regime is characterised by strong *jus sanguinis*, difficult naturalisation procedures and insistence on single citizenship (Hailbronner, 2002; Hailbronner & Farahat, 2015), implying that policy evolution is only possible through slow and gradual change (Green, 2004).

Further constraints on the evolution of the regime included, despite the recognition of having become an immigration country, the mobilisation of anti-immigrant public attitudes combined with tactical electoral considerations, which limited the room for reform to elite-level negotiations (Green, 2005; Howard, 2008). The SPD-Green coalition provided an opportunity in this respect, but their nationality law reform in 2000 fell short of expectations and did not result in a law as liberal as it was advertised (Green, 2012). But one remarkable development was the introduction of a model of *jus soli* conditional upon the renunciation of any other citizenship at the age of majority. It has taken yet another decade to introduce plural citizenship into the government agenda as a compromise for the ‘grand coalition’ formed after the 2013 federal election, and the 2014 reform brought about the tolerance of plural citizenship in the form of waiving the requirement of choosing only one citizenship. Although this last change is not a full endorsement of plural citizenship and only applies to those who are entitled to German citizenship as a birthright (Hailbronner & Farahat, 2015), it is eventually possible to be a dual citizen in Germany after a slow and gradual evolution of its citizenship regime.

## Emigration histories

### Historical context of emigration

#### Demographic, economic and political pressures in Mexico

Although emigration from Mexico dates back to the nineteenth century, the US Emergency Quota Act of 1921 constitutes a crucial turning point, as it exempted Mexicans (indeed Latin Americans) from immigration restrictions. However, Mexico was an under-populated country, hence the government was seeking to prevent any outflow of population (Fitzgerald, 2006a, 2009). Yet it was unable to do so due to the lack of control over borders on the part of Mexico and the welcoming of the crossers by the US. Nonetheless, in the following decades, population growth in Mexico made emigration a viable option for demographic management, coinciding with the labour shortage in the US due to the Second World War. Mexico, this time through bilateral agreements such as the Bracero Programme, had a better control over the flows. The underlying idea was to institutionalise migration as the supply of temporary workers. However, many migrants did not return and stayed in the US after the end of the programme in the mid-1960s, despite the disincentives for staying (such as not allowing family members) and the incentives for returning (such as 10% of the salary being conditional). Since the US was more willing to limit migration thereafter, Mexico had the flexibility to leave the burden of restrictions on the US, hence following the ‘policy of not having a policy’ (Martinez-Saldana, 2003).

The demographic concerns largely followed economic concerns, especially employment. In particular, the adoption of import substitution industrialisation (ISI) by Mexico as the development strategy was closely related to emigration becoming more viable, or even desirable (Canales, 2003). First, this strategy was partly responsible for the problem of not being able to create sufficient employment opportunities (Alba, 1978). Moreover, the basic logic being the construction of domestic industries which would produce goods that are normally imported, the orientation of ISI towards a protected internal market and reliance on the ability to import capital and intermediary goods entailed severe trade deficits (Hirschman, 1968). Thus, additional sources of foreign exchange inflow were needed and emigration could be seen as a channel of ‘migradollars’ in this regard. Although emigration could not solve the employment problem by itself, it was a temporary measure to mitigate its effects, and remittances were much appreciated.

Finally, the political situation creates an additional layer which shapes both the pressures for emigration and the government response. Broadly, the undemocratic character of the regime can be seen as causing not only politically motivated emigration by dissidents, but also an inability to reform the system and to develop successful migration policies (Martinez-Saldana, 2003). Yet the government stance on politically motivated migration also changed over time: while earlier concerns before the consolidation of regime were focused on opposition from abroad as a serious threat, later emigration came to be seen as exit option, hence a stabilising factor (Fitzgerald, 2006a, 2009).

#### Demographic, economic and political pressures in Turkey

Unlike Mexico, Turkey did not experience emigration as a challenge that aggravates under-population. Although the new republic was founded after a series of wars which lasted more than one decade and indeed led to severe under-population, demographic policies were focused on immigrants from former territories of the Ottoman Empire until population recovery in the 1940s. Meanwhile, freedom of travel was also legally restricted until 1961, and there was no need for additional measures in the absence of easily reachable attractive destinations among bordering or nearby countries. Thus, Turkey’s history of emigration starts directly with intergovernmental agreements with Germany (Abadan-Unat, 2011).

From this point onward, the similarities with Mexico’s experience after Bracero Programme are numerous. Migrants were initially expected to be temporary ‘guest’ workers (*Gastarbeiter*), but they became permanent residents and prepared the basis for further migration through family unification which was not allowed in the first place. When bilateral agreements ended as Germany sought to stop the inflow of migrants and encourage return due to 1973 Oil Crisis and ensuing stagflation and unemployment, Turkish government shifted to passive policies while German government increased restrictive measures. Illegal or uncontrolled migration also became an option, by going to Germany as tourists and settling there, usually with the help of relatives or acquaintances who had already migrated. Similarities continue as Turkey also adopted ISI as a development strategy. In line with ISI objectives, three major aims of the Turkish authorities in deciding to allow, encourage and organize migration were reducing unemployment, expanding foreign exchange reserves and upgrading human capital (Sayari, 1986). Although the objective of human capital upgrade through returning migrants was not met, temporary reduction of unemployment and increased foreign exchange inflow made positive contributions to development goals.

In terms of political pressures, Turkey differs from Mexico in that the single party rule established in Turkey in the 1920s did not last as long as PRI’s monopoly in Mexico, and emigration took place mostly in multi-party environments. However, due to several military interventions, regime consolidation was spread over decades, and an undemocratic character of the regime also shaped emigration. In this context, asylum seeking in Germany became common during the political turmoil of the 1970s and especially following the military intervention of 1980. While opposition from abroad was a relatively minor concern, politically motivated emigration gave the military regime the option to relieve political pressures by depriving the fleeing dissidents of their citizenship.

### Development of emigration state policies

#### Incentives, perceptions and demand for emigrant incorporation in Mexico

Once emigration occurs, ignoring emigrants is usually not a realistic option, and sending states develop specific policies, the motives for which primarily include securing economic resources, mobilising political support and providing protection and upward social mobility (Østergaard-Nielsen, 2003a). The last aspect may be either genuinely benevolent as expected from a state towards its citizens, or a way of further enhancing economic and political benefits that can be received. To begin with the economic sphere, following the above discussion on economic pressures, the obvious benefits consist of remittances, but these are not limited to ISI-related objectives since current account deficits have continued to be a serious challenge after the ISI period as well. Despite declining significance as a source of rural development over the last decade (Jones, 2014), the role of remittances in the Mexican economy in general has been remarkable up until today. According to World Bank data, the share of remittance inflows in GDP has shown an upward overall trend since the 1970s; more specifically, it was 0.53% in 1980, 1.18% in 1990, 1.10% in 2000, 2.10% in 2010 and 2.28% in 2015^6^. As a result, Mexico remains one of the top remittance-receiving countries today (World Bank, 2016).

Although some emigration is partially motivated by political dissidence, the resulting transnational links generate new opportunities for both domestic and international political objectives. In the domestic sphere, elections becoming more competitive and transparent in Mexico, regime legitimacy has become an increasingly pressing concern for which electoral support of emigrant communities has been crucial, ‘inadvertently helping create a transnational public sphere with greater democratic contestation’ (Smith, 2003b). In the international sphere, while foreign policy considerations certainly creates incentives for reaching out to emigrants, there is a serious risk of provoking receiving states by breaching non-intervention principle (Délano, 2009). Nonetheless, when the issue is cooperation between home and host countries, notably regional integration, conflict is less likely (Escobar, Hailbronner, Martin & Meza, 2006). During the formation process of NAFTA, Mexican government was more actively promoting the project and attributed an important role to emigrants to overcome the relatively reluctant attitude of the US (Goldring, 2002). This meant that the establishment of NAFTA played a central role in pushing Mexican government to embrace emigrant communities (Délano, 2009, 2011).

Despite these objective benefits, the views on emigrants in home countries can be quite negative especially in terms of social perceptions or cultural aspects. For instance, derogative labels such as *pochos* and *pachucos* are used for Mexican origin people living in the US to refer to their dissimilation from Mexican culture, assimilation to American culture, and indeed a subculture which is neither Mexican nor American. The legacy of Mexican experience with undesired emigration is reflected in the image of emigrants who abandoned their homeland. Yet, in later periods, negative views came to be replaced by the idea that they support their homeland from abroad, which is especially visible in the official discourse (Martinez-Saldana, 2003). Since this support was primarily in the form of economic contributions, cultural loss was outweighed by the economic gains. Thus, pursuing emigrant-friendly sending state policies has become socially more acceptable over time, in line with an improved image of emigrants.

In this context, several ethnographic studies (e.g. Besserer & Kearney, 2006; Gil, 2006; Ruvalcaba & Torres Robles, 2012) document intense transnational practices which link emigrants’ localities of origin and destination, and the central government’s role through programmes such as ‘Three for One’. Additionally, the greater involvement of the national government with hometown associations distinguishes Mexico from other Latin American countries (Portes, Escobar, & Radford, 2007). However, this does not mean that the government can ignore the political agency and autonomy of emigrants, and take their compliance for granted. In fact, critical attitudes towards Mexican government are prevalent, a considerable number of migrants show little interest in Mexican politics, and the basis of their actions will more likely be their interests but not abstract national attachments to the homeland (De la Garza & Desipio, 1998). Nor is it true that emigrants who are actively involved in transnational practices are those who remain culturally closer to the home country; on the contrary, integration into the host country seems quite compatible with transnational involvement (Portes et al., 2007).

Therefore, when emigrant support is sought for economic or political goals, either this should fall within an area of overlapping interests between emigrants and the sending state, or the latter should be able to offer something in return (Barry, 2006). In this respect, since unilateral policies cannot succeed alone, stronger economic and political incentives for reaching out to emigrants in the last decades brought about intensifying endeavours to institutionalise the links with them (Cano & Délano, 2007). On the one hand, emigrant organisations scattered across the US provided the opportunity of direct contact for the government, political parties and the Church; on the other hand, the government also sought to improve central coordination through the Institute of Mexicans Abroad (*Instituto de los Mexicanos en el Exterior*, established in 2003) (Fitzgerald, 2009).

More importantly, when the common interests of the Mexican government and emigrants are at stake, providing protection and upward social mobility becomes the crucial part of sending state policies. The nationality of home country naturally entails diplomatic protection and inalienable right to re-entry; on this front, for instance, Mexican consular officials succeeded in ensuring special treatment for undocumented Mexican migrants (Bakker, 2011). Beyond this, enabling the acquisition of host country citizenship empowers the emigrants to protect themselves, to improve their social status and to have political influence, while the most effective action for this purpose is accepting dual citizenship in the domestic legal system (Jones-Correa, 2001). In this respect, Mexico’s transition to a plural citizenship regime follows several anti-immigrant legislations in the US, such as California Proposition 187 of 1994, Welfare Reform Act and Illegal Immigration Reform and Immigrant Responsibility Act of 1996 (Castañeda, 2004; Escobar, 2006). In other words, the eventual acquiescence in plural citizenship can be linked to not simply the crystallising economic and political benefits or improving social perceptions, but also the intensifying institutionalisation of relations and the realisation of common interests which call for a better status in the host country.

#### Incentives, perceptions and demand for emigrant incorporation in Turkey

The main incentives for the Turkish governments to reach out to emigrants can also be understood in terms of economic and political benefits, and their overlapping interests to empower emigrants in Germany. In the economic dimension, similarly to Mexico, a particular importance was attached to remittances not only during ISI period, but also after the liberalisation of the economy towards an export-led model. In contrast to Mexico, the share of remittances in GDP has displayed a downward historical trend; more specifically, it was 3.01% in 1980, 2.16% in 1990, 1.67% in 2000, 0.24% in 2010 and 0.16% in 2015^7^. As a result, Turkey cannot be seen among the top remittance-receiving countries any more (World Bank, 2016). Nonetheless, it should be remarked that from the early 1980s into the mid-1990s when plural citizenship and blue card were legislated, the share of remittances was still considerably high, and even higher than that in Mexico.

In the dimension of international political objectives, Turkey’s bid to become a member of the EU is comparable to Mexico’s interest in NAFTA, with several major differences. Turkey is trying to accede to an advanced level of regional integration; both the conditions of accession are more demanding, and a possible future membership would have more serious economic and political implications. When accession is high in Turkish political agenda, emigrants might have invaluable influence on the attitude of current members whose approval is indispensable, and in particular Germany, the biggest member of the EU (Østergaard-Nielsen, 2003d,e). Furthermore, EU membership has direct implications for citizenship; if Turkish emigrants in Germany were classified as EU citizens, they would be subject to privileged treatment for dual citizenship without the requirement of renouncing their existing nationality. In other words, the effect of Turkey’s accession to the EU on plural citizenship would be equivalent to convincing Germany to amend her nationality law.

As for social perceptions of emigrants, similar forms of contempt exist in Turkish society as well; the term *almancı* usually carries derogative connotations, and the subculture of emigrants are seen as traditional, backward, degenerative, or at best ‘in-between’ (Kaya, 2005). As different from the perception of Mexican-Americans, however, an element of betrayal is not reflected in these views. The official counterpart of this has been expectations from migrants to represent modern and secular Turkey, as opposed to their traditional backgrounds (Østergaard-Nielsen, 2003e). However, approaching the identity of migrants with modern-traditional dichotomy is a deep misconception; Turkish emigrants in Germany and other countries of the EU display a hyphenated identity that suggests identification with Turkey, Germany and Europe at the same time, and transnational practices that cannot be limited to any of the national spheres (Kaya, 2012). In this sense, a possible accession to the EU would not only serve their interests, but also affirm their identities, and unsurprisingly their support for this goal is strong (Kaya & Kentel, 2005).

Nevertheless, this is one of the few areas in which Turkish authorities can receive large support. Otherwise, the Turkish community in Germany is deeply divided along party-political, ethnic and religious lines, replicating the divisions in Turkey, carrying the political fragmentation of Turkey into German public sphere, and resulting in a high number of associations representing diverse views and interests (Ögelman, 2003; Østergaard-Nielsen, 2003d; Yurdakul, 2006). In order to deal with this diversity, Turkey followed a similar path as Mexico, and established central coordination offices which are more recently structured into the Secretariat of Turks Abroad and Kin Communities (*Yurtdisi Türkler ve Akraba Topluluklar Baskanligi*, established in 2010), paying special attention to extending the continuous contact with Turkish NGOs in Germany.

Probably the most interesting aspect of the Turkish case is that the acceptance of dual citizenship, codified in 1981, precedes all these institutional attempts. At that time, even the EU accession was at best a vague and distant goal which could not directly result in such a radical change. However, it can be presumed that Turkey has always had a foreign policy oriented towards Europe, and a strong interest in making itself accepted as a European country. In the subsequent decades, as Turkey developed an even stronger and a more concrete interest in creating sympathy for herself in German politics, her approach to plural citizenship also evolved (Kadirbeyoglu, 2009) from passive acceptance to active promotion, and to expressing dissatisfaction with German insistence on single citizenship.

## Discussion

The above comparison is intended to reveal reasons behind diverging preferences of emigration states regarding plural citizenship. Before this, however, the overall conception of nationhood and citizenship in the examined countries, as laid out in Citizenship constellations section, constitutes the background against which any reform occurs, irrespectively of the experience with emigration. As seen in immigration countries as well, such conceptions and the institutions that are built upon them have a resilient nature. In this sense, the territorial conception in Mexico is biased against the absent nationals, especially those born abroad, while the ethno-cultural conception in Turkey prioritises transnational ties. Although this can be seen as a sufficient explanation for diverging preferences, there is still a need to explain how the path-dependence of the Mexican regime was altered, and why the Turkish authorities not only accepted, but also actively promoted the acquisition of German or any other foreign citizenship. For this purpose, the below discussion will explore how emigration histories are linked to the timing of major citizenship regime reforms in each case.

Since the comparison of Mexico and Turkey is designed as one of similar systems, inferences can be primarily derived from their differences. In terms of the historical context, the main difference lies in the early phases of emigration from Mexico. In the time period after the mid-XX^th^ century, the two countries display highly similar features: controlled migration through bilateral agreements, which was desirable for development objectives and unchallenging in demographic terms, unfulfilled expectations about return migration, dilemmas of politically motivated emigration, among others. In the preceding period when there was no emigration from Turkey, Mexico’s experience was characterised by undesirable and demographically challenging emigration, which led to a historical legacy of scepticism towards emigrants, with several forms of contempt in the society and even a sense of being betrayed. Therefore, this difference in the emigration history constitutes a likely explanation as to why Mexico was so late to fully endorse emigrant communities. However, in the absence of a negative image of emigrants during later phases of emigration, even a socially entrenched scepticism would naturally fade away over time, especially as the society undergoes a generational change. In this sense, the reform of the citizenship regime in 1997 can be seen as coinciding with a time when a negative image of emigrants was no longer significant enough to influence political decisions. On the contrary, in the case of Turkey, the absence of such negative experiences meant that the full endorsement of expatriates was unproblematic, and plural citizenship could be legislated as soon as it was realised that emigrants had become permanent residents of another country, namely in the early 1980s.

As for the factors which led Mexico and Turkey to develop sending state policies, they differ in the relative importance of economic and political benefits. A closer look into the economic factors, however, reveals that the different trends cannot be directly associated with diverging citizenship regime preferences. Namely, the remittance inflow displays, since 1980, an upward trend in Mexico and a downward trend in Turkey. Yet, during the period leading to the reform in Mexico, remittances had remained a considerable, and more importantly, consistent source of foreign exchange at approximately 1.00% of GDP. In Turkey, the share of remittances had been higher than Mexico in the 1980s and 1990s, when relevant citizenship reforms occurred, but a downward trend was already apparent. A more striking difference is visible in the early 2000s, with a sharp increase in Mexico and a sharp decrease in Turkey. Although this was after major citizenship reforms, it should be noted that Turkey continued the efforts to extend external voting in this period when the share of remittances in GDP was consistently low around 0.20%. In this context, the differences in the level and the trend of economic benefits do not seem to align with the preferences for dual citizenship regimes. One likely explanation is that citizenship is more relevant for legal and political aspects of the relationship between emigrants and the country of origin, while economic contributions do not necessarily require formal ties.

The differences between Mexico and Turkey regarding political incentives can thus be associated with citizenship regime reforms, since the dual citizenship of emigrants is more relevant for achieving political goals. Regarding the persistence of home politics among emigrants, domestic issues of Turkey have been playing an important role in the lives of Turkish communities abroad, while there is at best mixed evidence for similar patterns in the case of Mexicans. When regional integration initiatives are considered, the accession of Turkey to the EU, albeit less likely, is a stronger incentive which has been sustained over time. This goal entered the political agenda even before the acceptance plural citizenship and it has not been dropped since then. Even though it may be too bold to claim that the 1981 reform was made with EU accession in mind, it can still be understood more broadly as part of Turkey’s political orientation towards the West, especially under Cold War conditions. In the case of Mexico, NAFTA does not represent a degree of regional integration comparable to the EU, and the place of such an integration in the political agenda has not always been as high as in the 1990s. In addition, regarding other political issues as discussed above, the anti-immigrant legislations in the US in the mid-1990s can also be counted among factors which contributed to the decision to reform the citizenship regime. In this sense, although Mexico did not have long-standing political incentives like Turkey, the intensifying motives during the early and mid-1990s seem to be the reasons why the reform took place in this period. In other words, political motives emerge as a factor strong enough to alter the path-dependence of the Mexican citizenship regime.

In sum, the experience of Mexico with undesirable emigration in the early phases of her migration history can be seen as one of the reasons why a reform towards dual citizenship was so delayed. Meanwhile, there were not sufficiently strong political incentives for a long time, and the economic incentives did not seem to have played a crucial role. Nonetheless, the 1990s witnessed an improved image of emigrants and the culmination of several political factors which led to the citizenship reform. It was this delay in the transition to a plural citizenship regime that created an incompatibility with the liberal regime of the US, and during this time Mexicans living abroad enjoyed only a limited status of nationality, or a status of quasi-citizenship. In the case of Turkey, the absence of a historical legacy of undesirable migration and the presence of long-standing strong political incentives facilitated an early transition to the plural citizenship regime. However, due to Germany’s insistence on single citizenship, the dual goal of retaining ties with the Turkish communities abroad and increasing their political influence necessitated a category which does not entail a legal status of nationality, hence a quasi-citizenship in the form of pink/blue card.

Although both constellations ended up in regimes which allow the practice of dual citizenship, the limits should also be noted. Mexico institutionalised plural citizenship together with a restriction on its transmission abroad. Without this last provision, it was unable to know through ordinary means who had been naturalised into US citizenship, that is, to ensure full compliance with a single citizenship rule. The reform allows dual citizenship, but also prevents an uncontrollable expansion of absent citizens. In this sense, it is as much a response to the need for plural citizenship as a response to the inefficiency of resisting it. Hence, if unlimited transmission to future generations is considered an important aspect, external and internal citizenships of Mexico are still not perfectly equivalent. On the other side, Germany tolerates plural citizenship by waiving certain conditionalities, but without explicitly recognising it. First, this means that those who are not entitled to German citizenship as a birthright still constitute a considerable group of potential quasi-citizens. Second, this was due to a political compromise necessary for a coalition government to be formed, and it is yet to be seen whether a changing balance of power will cause a setback to the previous system or to a full endorsement. Therefore, the incompatibility between Turkish and German regimes continues to a certain extent since plural citizenship is not fully institutionalised in the latter, and in the case of a reversal, quasi-citizenship can once again become a relevant category for an even larger group and younger generations.

## Conclusion

This paper has conceptualised quasi-citizenship as a category less extensive than full citizenship in terms of its constituent aspects, but which partially performs functions which are traditionally associated with citizenship. While resident non-citizens, or denizens, provide a familiar example, the focus has been on the forms of emigrant quasi-citizenship. It has been argued that emigrant quasi-citizenship is a transitional equilibrium on the path to plural citizenship, emerging from an incompatibility of sending and receiving state preferences regarding the latter. Such preferences of the sending states depend on their experience with emigration. The comparison of Mexico and Turkey reveals that the historical legacy of past undesirability of emigration can result in an insistence on single citizenship, while higher relevance of citizenship for international political goals lead not only to swift acceptance of dual citizenship, but also to the active promotion of the acquisition of host country citizenship. Consequently, where the sending state pulls the equilibrium away from plural citizenship, quasi-citizenship takes the form of curtailing the existing category of citizenship, and where the sending state pushes the equilibrium towards plural citizenship, quasi-citizenship takes the form of inventing new statuses.

Although the findings of this analysis are not readily generalisable, several implications can be put forward for consideration in broader citizenship and migration studies. To begin with, the reciprocal preferences of immigration and emigration states resulted in different trajectories of quasi-citizenship which shaped the legal status and the political rights of large groups of migrants for a long period of transnational migration. In this sense, the transition to the plural citizenship regime is not straightforward, even though the global trend is apparently in this direction. The case of Mexico shows that the economic asymmetry between the sending and receiving states, which underpins the transnational labour migration, does not directly translate into an instrumentalisation of citizenship for emigrant incorporation. The case of Germany shows that the full institutionalisation of plural citizenship does not automatically follow the recognition of having become an immigration country, even if the liberalisation of the citizenship regime is already underway.

Another implication is concerned with the relationship between sending state policies and different conceptions of national citizenship. The case of Mexico shows that it is possible to embrace emigrants as full citizens and accept dual citizenship without fundamentally re-ethnicising the regime. In fact, the reform in Mexico which institutionalised plural citizenship also introduced a new limit on the non-territorial transmission of citizenship. In the case of Turkey, the strong ethno-cultural character of the citizenship regime is probably one of the primary reasons why Turkish authorities were so keen on plural citizenship so early. In this sense, an ethno-cultural conception of citizenship may be a sufficient condition for accepting plural citizenship in the presence of significant numbers of emigrants, yet it cannot be seen as a necessary condition as it is possible to reconcile plural citizenship with a territorial conception.

Furthermore, two major variables emerged from the above comparative study as potential explanations for the preferences of emigration states: the historical legacy of emigration and the strength of political motives. For the first, it has been suggested that the legacy of undesirable emigration and the ensuing negative image of emigrants might have been a preventing factor for the development of policies favourable towards plural citizenship. In this sense, for the emigration state to embrace emigrants as full citizens, hence to implement plural citizenship policies, it seems necessary that the migrants are socially deemed as worthy of home country citizenship. Second, it has been suggested that citizenship is particularly relevant for attaining political objectives which can significantly facilitate or accelerate the transition to a plural citizenship regime. In this sense, for such a reform to occur, it seems sufficient that the strength of political motives outweighs any preventing factor or the cost of departing from institutional path-dependence.

Following the last point, the objective value of citizenship remains high as long as it is a precondition for achieving certain political goals, either for the migrants or for the states. With regard to sending state policies, the citizenship of the country of origin is necessary for maintaining formal ties and expanding domestic politics into the transnational sphere, and the citizenship of the country of residence is necessary for a potential assistance by emigrants in pursuing international political objectives. Therefore, emigration states can be expected to promote or retain plural citizenship regimes, although immigration country governments may be sceptical about their political implications. In the case of Mexico-US constellation, the current controversies are mostly at the policy-level, while the citizenship regimes have firm constitutional bases in both countries, hence they are unlikely to change soon. The constellation that requires continuous scholarly attention is that of Turkey-Germany, for reasons related to both countries.

As stated earlier, the toleration of dual citizenship in Germany did not result from its full institutionalisation, but from a political compromise. In this sense, it is still sensitive to political dynamics in the country, and migration was one of the most contentious issues for the recent federal election which has witnessed the rise of an anti-immigration party. Meanwhile, Turkey’s drift away from the goal of EU accession does not mean a decrease in the political relevance of emigrants, but the government has not recently been following policies which are likely to create sympathy for German-Turks. Recent examples include the controversy around the constitutional referendum as a repercussion of the democratic erosion in Turkey, and an attempt by the Turkish president to influence the votes cast by Turkish-origin citizens in the last federal election. Both lines of transnational politics create serious doubts about the dual citizenship of Turkish-origin migrants in a fragile political environment.

## Endnotes

^1^ A dimension of active citizenship can also be added. But this usually refers to ex-post questions about the duties of citizens once the citizenry is more or less known. The other three dimensions are more relevant for ex-ante questions regarding who is a citizen.

^2^ New Zealand comes forth as the main exception to this rule. Further exceptions can be found elsewhere usually for local elections, for example within the EU, or for a specific group of immigrants, such as Commonwealth citizens in the UK.

^3^ Significant migrant communities also exist in other European countries, notably France, Belgium, the Netherlands, Switzerland, etc. However, Germany is by far the most popular and the oldest destination, the numbers of Turkish-origin people living in Germany and other European countries today are not comparable, and Germany occupies a particularly important place for Turkish migration policies.

^4^ The author’s translation from the Political Constitution of the Mexican United States (Constitución Política de los Estados Unidos Mexicanos).

^5^ Andrés Bermúdez Viramontes was a successful tomato rancher living in California, hence the name ‘Tomato King’. The controversy was caused by a court decision which invalidated his first election as a mayor of Jerez, Zacatecas in 2001. Yet he was re-elected in 2003, and seen as a symbol of the opening of Mexican politics to migrants (Smith & Bakker 2005).

^6^ These percentages are calculated from the data reported by the World Bank on its official website: https://data.worldbank.org/ (access: 28/09/2017). The indicators used in these calculations are ‘Personal remittances, received (current US$)’ and ‘GDP (current US$)’.

^7^ These percentages are also calculated from the data reported by the World Bank on its official website: https://data.worldbank.org/ (access: 28/09/2017). The indicators used in these calculations are ‘Personal remittances, received (current US$)’ and ‘GDP (current US$)’.
